# Involvement of nitric oxide synthase in matrix metalloproteinase-9- and/or urokinase plasminogen activator receptor-mediated glioma cell migration

**DOI:** 10.1186/1471-2407-13-590

**Published:** 2013-12-11

**Authors:** Thompson Zhuang, Bharath Chelluboina, Shivani Ponnala, Kiran Kumar Velpula, Azeem A Rehman, Chandramu Chetty, Eleonora Zakharian, Jasti S Rao, Krishna Kumar Veeravalli

**Affiliations:** 1 Department of Cancer Biology and Pharmacology, University of Illinois College of Medicine at Peoria, One Illini Drive, Peoria, IL 61605, USA; 2 Department of Neurosurgery, University of Illinois College of Medicine at Peoria, Peoria, IL 61605, USA

**Keywords:** Glioma, Nitric oxide, Migration, Integrin, Knockdown

## Abstract

**Background:**

Src tyrosine kinase activates inducible nitric oxide synthase (iNOS) and, in turn, nitric oxide production as a means to transduce cell migration. Src tyrosine kinase plays a key proximal role to control α9β1 signaling. Our recent studies have clearly demonstrated the role of α9β1 integrin in matrix metalloproteinase-9 (MMP-9) and/or urokinase plasminogen activator receptor (uPAR)-mediated glioma cell migration. In the present study, we evaluated the involvement of α9β1 integrin-iNOS pathway in MMP-9- and/or uPAR-mediated glioma cell migration.

**Methods:**

MMP-9 and uPAR shRNAs and overexpressing plasmids were used to downregulate and upregulate these molecules, respectively in U251 glioma cells and 5310 glioma xenograft cells. The effect of treatments on migration and invasion potential of these glioma cells were assessed by spheroid migration, wound healing, and Matrigel invasion assays. In order to attain the other objectives we also performed immunocytochemical, immunohistochemical, RT-PCR, Western blot and fluorescence-activated cell sorting (FACS) analysis.

**Results:**

Immunohistochemical analysis revealed the prominent association of iNOS with glioblastoma multiforme (GBM). Immunofluorescence analysis showed prominent expression of iNOS in glioma cells. MMP-9 and/or uPAR knockdown by respective shRNAs reduced iNOS expression in these glioma cells. RT-PCR analysis revealed elevated iNOS mRNA expression in either MMP-9 or uPAR overexpressed glioma cells. The migration potential of MMP-9- and/or uPAR-overexpressed U251 glioma cells was significantly inhibited after treatment with L-NAME, an inhibitor of iNOS. Similarly, a significant inhibition of the invasion potential of the control or MMP-9/uPAR-overexpressed glioma cells was noticed after L-NAME treatment. A prominent reduction of iNOS expression was observed in the tumor regions of nude mice brains, which were injected with 5310 glioma cells, after MMP-9 and/or uPAR knockdown. Protein expressions of cSrc, phosphoSrc and p130Cas were reduced with simultaneous knockdown of both MMP-9 and uPAR.

**Conclusions:**

Taken together, our results from the present and earlier studies clearly demonstrate that α9β1 integrin-mediated cell migration utilizes the iNOS pathway, and inhibition of the migratory potential of glioma cells by simultaneous knockdown of MMP-9 and uPAR could be attributed to the reduced α9β1 integrin and iNOS levels.

## Background

High grade gliomas invariably recur due in a large part to tumor cells penetrating the normal brain in an inaccessible, diffuse manner. Further, the tendency of glioblastoma multiforme (GBM) cells to migrate and invade normal brain tissue renders surgical interventions ineffective [1]. Glioma cell migration and invasion is generally separated into three phases. First, the glioma cells attach to proteins located in the extracellular matrix (ECM) with the aid of cell adhesion receptors. Subsequently, ECM proteins are degraded by proteases secreted by the glioma cells, such as MMPs and serine proteases. ECM degradation provides opportunity for active glioma cell migration through the intercellular space. In human glioma cells, MMP-9 and uPAR have been found to be overexpressed. MMP-9 has been implicated in ECM degradation, angiogenesis, and subsequent tumor growth and invasion [2,3]. A strong relationship exists between MMP-9 levels and cell migratory/invasive potential due to the crucial role of MMPs in proteolysis of the ECM. Of the MMPs, MMP-9 was found to be most closely linked to tumor grade [4-7]. In addition to MMPs, the serine protease uPA has been established to be active in the degradation of the ECM. The binding of uPA to uPAR is essential both *in vitro* and *in vivo* for cancer cell metastasis, invasion, and migration. Inhibition of uPAR prevented cancer cell metastasis. Elevated levels of both uPA and uPAR were observed in human carcinoma cells, elucidating uPAR’s critical role in cancer cell migration. Silencing MMP-9 and/or uPAR decreased cell adhesion to ECM proteins—a process known to promote tumor cell migration and invasion [8]. MMP-9 and/or uPAR gene silencing also reduced invasive/migratory potential and growth of glioma cells [8]. Our recent studies clearly demonstrated the involvement of α9β1 integrin in MMP-9-/uPAR-mediated glioma cell migration [9]. Integrin α9β1 regulates inducible nitric oxide synthase (iNOS) activity via Src tyrosine kinase; Src coordinates subsequent signaling pathways through activation of FAK and tyrosine phosphorylation of the adaptor protein p130Cas [10].

Inducible nitric oxide synthase and nitric oxide (NO) are closely linked to tumor growth, proliferation, and poor prognosis in humans with malignant glioma. NO is a heme co-factor that activates soluble guanylyl cyclase (GC) to produce cGMP, which regulates cell migration in both a protein kinase G (PKG) dependent and independent fashion [11,12]. NO, derived from tumor iNOS, is an important modulator of tumor progression and angiogenesis in C6 glioma cells [13]. Tumor-derived NO may also promote invasiveness through the induction of MMP-9 expression by tumor cells. Tumors with MMP-9 overexpression had significantly higher iNOS activity and cGMP levels compared with tumors that had absent or focal expression of MMP-9 in head and neck squamous cell carcinoma [14]. Recently, it was reported that α9β1 integrin regulates iNOS activity, which resulted in increased NO production and NO-induced cell migration [10]. Because α9β1 integrin plays a crucial role in MMP-9 and uPAR-mediated cell migration in glioma, we hypothesized that MMP-9 and uPAR utilize iNOS via α9β1 integrin to arbitrate cell migration. In the present study, we investigated the involvement of the α9β1 integrin-iNOS pathway in MMP-9- and/or uPAR- mediated glioma cell migration.

## Methods

### Ethics statement

The Institutional Animal Care and Use Committee of the University of Illinois College of Medicine at Peoria, Peoria, IL approved all surgical interventions and post-operative animal care.

### Chemicals and reagents

L-N^G^-Nitroarginine methyl ester (L-NAME) was obtained from Sigma (St. Louis, MO). Recombinant human uPAR was obtained from R&D Systems (Minneapolis, MN). Anti-α9β1 integrin, anti-NOS2, anti-cSRC and anti-p130Cas antibodies were obtained from Santa Cruz Biotechnology (Santa Cruz, CA). Anti-phosphoSRC (Tyr 416) antibody was obtained from Cell Signaling (Boston, MA). Anti-glyceraldehyde-3-phosphate dehydrogenase (GAPDH) antibody was obtained from Novus Biologicals (Littleton, CO). Diaminofluorescein-2 Diacetate (DAF-2DA) was obtained from Enzo Life Sciences (Farmingdale, NY).

### Construction of shRNA- and gene-expressing plasmids

Plasmid shRNAs for MMP-9 (M-sh), uPAR (U-sh) and MMP-9-uPAR (MU-sh) were designed in our laboratory [15] and used to transfect the cells. Briefly, a pCDNA-3 plasmid with a human cytomegalovirus (CMV) promoter was used to construct the shRNA-expressing vectors. A pCDNA3-scrambled vector with an imperfect sequence, which does not form a perfect hairpin structure, was used as a control (SV-sh). MMP-9 human cDNA cloned in pDNR-CMV vector in our laboratory was used for full-length MMP-9 (M-fl) overexpression. We used uPAR human cDNA cloned in pCMV6-AC vector (Origene, Rockville, MD) for full-length uPAR (U-fl) overexpression.

### Cell culture and transfection conditions

U251 human glioma cells obtained from the National Cancer Institute (NCI) (Frederick, MD) were grown in DMEM supplemented with 10% fetal bovine serum (FBS) (Hyclone, Logan, UT) and 1% penicillin/streptomycin (Invitrogen, Carlsbad, CA). 5310 human glioma xenograft cells were kindly provided by Dr. David James at the University of California, San Francisco. These xenografts were generated and maintained in mice and are highly invasive in the mouse brain [16]. 5310 xenografts were maintained in RPMI 1640 supplemented with 10% fetal bovine serum and 1% penicillin/streptomycin at 37°C in a humidified atmosphere containing 5% CO_2_. U251 and 5310 cells were transfected with SV-sh, M-sh, U-sh, MU-sh, M-fl, or U-fl using Fugene® HD reagent obtained from Roche Diagnostics, (Indianapolis, IN) according to the manufacturer’s instructions.

### Wound healing assay

To study cell migration, we seeded U251 glioma cells at a density of 1.5 × 10^6^ or 2 × 10^6^ in a 6-well plate and transfected the cells with M-fl, or U-fl for 72 hrs. Then, a straight scratch was made in individual wells with a 200 μl pipette tip. This point was considered the “0 hr,” time point and the width of the wound was photographed under the microscope. Again at the 21^st^ hr, the cells were checked for wound healing and photographed under the microscope. Wound healing was measured by calculating the reduction in the width of the wound after incubation. The involvement of the iNOS pathway on M-fl- or U-fl-mediated glioma cell migration was assessed by adding L-NAME (1 mM final concentration) at “0 hr” to the appropriate wells containing glioma cells transfected with M-fl, or U-fl.

### Spheroid migration assay

U251 glioma cells were cultured in 96-well plates coated with 1% agar. Briefly, 3 × 10^4^ cells/well were seeded and cultured on a shaker at 100 rpm for 48 hr in a humidified atmosphere containing 5% CO_2_ at 37°C. After the formation of spheroids, they were transfected with M-fl or U-fl overexpressing plasmids. 48 hr after transfection, the spheroids were transferred to 24-well plates at a density of one spheroid/well and incubated at 37°C. At this time point, a few spheroids from each group were treated with L-NAME at a final concentration of 1 mM. Twenty-four hours after incubation, the spheroids were fixed and stained with Hema-3. Cell migration from the spheroids was assessed using light microscopy. The migration of cells from spheroids to monolayers was used as an index of cell migration and was measured using a microscope calibrated with a stage and ocular micrometer.

### Matrigel invasion assay

U251 and 5310 glioma cells were transfected with M-fl or U-fl for 72 hr. Cells were trypsinized and 5 × 10^4^ cells were placed onto Matrigel-coated transwell inserts of 8-mm pore size. A few of the transwells containing untreated and M-fl- or U-fl-transfected glioma cells were then subjected to L-NAME (1 mM) treatment. Cells were allowed to migrate through the Matrigel for 24 to 48 hr. Then, cells in the upper chamber were removed with a cotton swab. The cells that adhered on the outer surface of the transwell insert and had invaded through the matrigel were fixed, stained with Hema-3, and counted under a light microscope as described earlier (Veeravalli et al., [8]).

### Intracranial administrations in nude mice

5310 glioma xenograft cells were trypsinized and re-suspended in serum-free medium at a concentration of 0.2 × 10^5^ cells/μL. Nude mice were injected intracerebrally with 10 μL aliquot (0.2 × 10^5^ cells/μL) under isofluorane anesthesia with the aid of a stereotactic frame. After two weeks, mice were separated into four groups. The first group served as control. The second, third, and fourth groups served as M-sh-treated (150 μg), U-sh-treated (150 μg), and MU-sh-treated (150 μg) groups, respectively. M-sh, U-sh and MU-sh plasmid DNAs were injected into the brains of nude mice using Alzet mini pumps at the rate of 0.2 μL/hr. The concentration of the plasmid solution was 2 μg/μL (100 μl per mouse, six mice in each group). After 5 weeks, the mice were sacrificed by intracardiac perfusion, first with PBS and then with 4% paraformaldehyde in normal saline. The brains were removed, stored in 4% paraformaldehyde, processed, embedded in paraffin, and sectioned (5 μm thick) using a microtome. Paraffin-embedded sections were processed for immunohistochemical analysis.

### Immunohistochemical analysis

Paraffin-embedded brain sections (5 μm thick) from control and treatment groups were de-paraffinized following standard protocol. The sections were rinsed with PBS and treated with 1% BSA in PBS to prevent non-specific staining and incubated with anti-iNOS antibody (1:100 dilution) at 4°C overnight. The sections were then washed in PBS and incubated with the appropriate HRP-conjugated secondary antibody for 1 hr at room temperature. After 1 hr, the sections were washed in PBS and incubated in DAB for 30 min. The slides were further washed with sterile water, stained with hematoxylin and dehydrated. The slides were then covered with glass cover slips and photomicrographs were obtained. Immunohistochemical analysis for iNOS protein expression was also performed on the slide tissue microarrays (obtained from US Biomax, Inc., Rockville, MD) of clinical GBM samples according to the manufacturer’s instructions.

### Immunocytochemical analysis

U251 and 5310 cells (1 × 10^4^) were seeded on 2-well chamber slides, incubated for 24 h, and transfected with SV-sh, M-sh, U-sh, or MU-sh for 72 hrs. Then, cells were fixed with 10% buffered formalin phosphate and incubated with 1% bovine serum albumin in PBS at room temperature for 1 hr to avoid non-specific staining. After the slides were washed with PBS, anti-iNOS antibody was added at a concentration of 1:100. The slides were incubated overnight at 4°C and washed three times with PBS to remove excess primary antibody. Cells were then incubated with Alexa Fluor® 594 (goat anti-mouse IgG, red) fluorescent-labeled secondary antibody for 1 hr at room temperature. The slides were then washed another three times with PBS, exposed to DAPI containing mounting media, covered with glass coverslips, and fluorescent photomicrographs were obtained.

### Reverse transcription PCR analysis

Total cell RNA was isolated from untreated U251 and 5310 glioma cells and from those transfected with M-fl, or U-fl. Approximately 1 μg of total RNA from each sample was synthesized into cDNA following the manufacturer’s instructions using the Transcriptor First Strand cDNA Synthesis Kit obtained from Roche Diagnostics (Indianapolis, IN). We used the following sequences for the forward and reverse primers:

• for iNOS, 5′cgqiztgtggaagcggtaacaaagga3′ (forward) and 5′tgccattgttggtggagtaa3′ (reverse);

• for βActin, 5′ggcatcctcaccctgaagta3′ (forward) and 5′ggggtgttgaaggtctcaaa3′ (reverse).

Reverse transcription - polymerase chain reaction (RT-PCR) was set up using the following PCR cycle: 95°C for 5 min, (95°C for 30 sec, 55–60°C for 30 sec, and 72°C for 30 sec) × 35 cycles, and 72°C for 10 min. PCR products were resolved on a 1.6% agarose gel, visualized, and photographed under UV light.

### Western blot analysis

U251 and 5310 cells were transfected with SV-sh, M-sh, U-sh, M-fl and U-fl for 72 hrs. Cells were collected and lysed in RIPA buffer [50 mmol/mL Tris–HCl (pH 8.0), 150 mmol/mL NaCl, 1% IGEPAL, 0.5% sodium deoxycholate, 0.1% SDS] containing 1 mM sodium orthovanadate, 0.5 mM PMSF, 10 μg/mL aprotinin, 10 μg/mL leupeptin and resolved via SDS-PAGE. After overnight transfer onto nitrocellulose membranes, blots were blocked with 5% non-fat dry milk in PBS and 0.1% Tween-20. Blots were then incubated with primary antibody, followed by incubation with HRP-conjugated secondary antibody. Immunoreactive bands were visualized using chemiluminescence ECL Western blotting detection reagents on Hyperfilm-MP autoradiography film obtained from Amersham (Piscataway, NJ). GAPDH (housekeeping gene) antibody was used to verify that similar amounts of protein were loaded in all lanes.

### FACS analysis

U251 and 5310 cells were seeded on 100-mm tissue culture plates. Cells were transfected with M-fl, transfected with M-fl and blocked with α9β1 antibody, treated with recombinant uPAR or treated with recombinant uPAR and blocked with α9β1 antibody. 48–72 hrs after transfection or 1–2 hrs after recombinant uPAR treatment, cells were treated with 50 mM EDTA, washed with PBS, pelleted at 1000 rpm for 5 min, and re-suspended in PBS in an appendorff tube at a concentration of 1 × 10^6^ cells/mL. Cells were then incubated with HRP-conjugated iNOS antibody for 1 hr on ice, pelleted, and washed three times with PBS to remove excess primary antibody. Cells were then re-suspended in 1 ml of PBS and incubated with Alexa Fluor® 594 (goat anti-mouse IgG, red) fluorescent labeled secondary antibody for 1 hr on ice. After three more washes in PBS, cell pellet was re-suspended in 10% buffered formalin and analyzed on a Coulter EPICS XL AB6064 flow cytometer (Beckman Coulter, Fullerton, CA).

### Detection of NO in 5310 glioma cells

DAF-2DA is a non-fluorescent cell permeable reagent that can measure free NO in living cells. Once inside the cell, the diacetate groups of the DAF-2DA reagent are hydrolyzed by cytosolic esterases, thus releasing DAF-2 and sequestering the reagent inside the cell. Production of NO in the cell, if any, converts the non-fluorescent dye, DAF-2, to its fluorescent triazole derivative, DAF-2 T. 5310 glioma xenograft cells cultured in 12-well plates were transfected with MMP-9 or uPAR overexpressing plasmids (M-fl or U-fl, respectively) or MU-sh plasmid shRNA. Seventy two hours after transfection, a few wells containing M-fl or U-fl transfected 5310 cells were treated with L-NAME (1 mM). In order to demonstrate that MMP-9 and uPAR-mediated glioma cell migration utilizes nitric oxide, four hours after treatment with L-NAME, 5310 glioma cells from all the treatment groups including controls were treated with DAF-2DA reagent and the cells were incubated for 60 min at 37°C. To remove the excess dye and stain, the nucleus for quantitative analysis, samples were washed with PBS and resuspended in PBS containing DAPI. Green fluorescence and the respective DAPI images were captured by using a fluorescent microscope.

### Densitometry

Densitometry was performed using Image J Software (National Institutes of Health) to quantify the band intensities obtained from Western blot analysis. Data represent average values from three separate experiments.

### Statistical analysis

Statistical comparisons were performed using Graph Pad Prism software (version 3.02). Quantitative data from Western blot analysis, wound healing assay, spheroid migration assay and matrigel invasion assays were evaluated for statistical significance using one-way ANOVA. Bonferroni’s post hoc test (multiple comparison tests) was used to compare any statistical significance between groups. Differences in the values were considered significant at p < 0.05.

## Results and discussion

### Effect of inhibition of iNOS on cell migration and invasion

**Figure 1:**
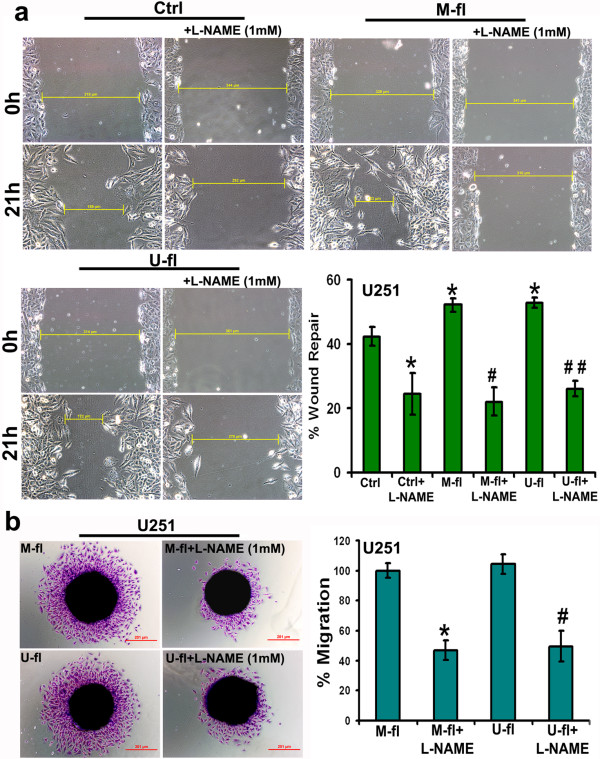
**Migration potential of U251 glioma cells reduced after treatment with iNOS inhibitor. (a)** U251 glioma cells were cultured in six-well plates and transfected with full-length MMP-9 (M-fl) and uPAR (U-fl) plasmids. 72 hrs after transfection, a straight scratch was made in individual wells with a 200 μL pipette tip. This point was considered to be the 0 hr, and the width of the wound was photographed under a microscope. At this point, additional wells of a six-well plate containing U251 cells from control, M-fl and U-fl treatments were subjected to treatment with L-NAME, an inhibitor of iNOS at 1 mM concentration. At the 21^st^ hr, the cells were checked for wound healing and again photographed under a microscope. Bar graph represents the quantification of wound healing assay results. Columns represent mean (n = 3). Error bars represent ± SEM. *p < 0.05 vs. control (Ctrl). ^#^*p* < 0.05 vs. M-fl. ^##^*p* < 0.05 vs. U-fl. **(b)** U251 spheroids were transfected with M-fl and U-fl plasmids. A few spheroids from each group were treated with L-NAME. Bar graph represents the quantification of cell migration from the spheroids. Columns represent mean (n = 3). Error bars represent ± SEM. *p < 0.05 vs. M-fl. ^#^*p* < 0.05 vs. U-fl.

**Figure 2:**
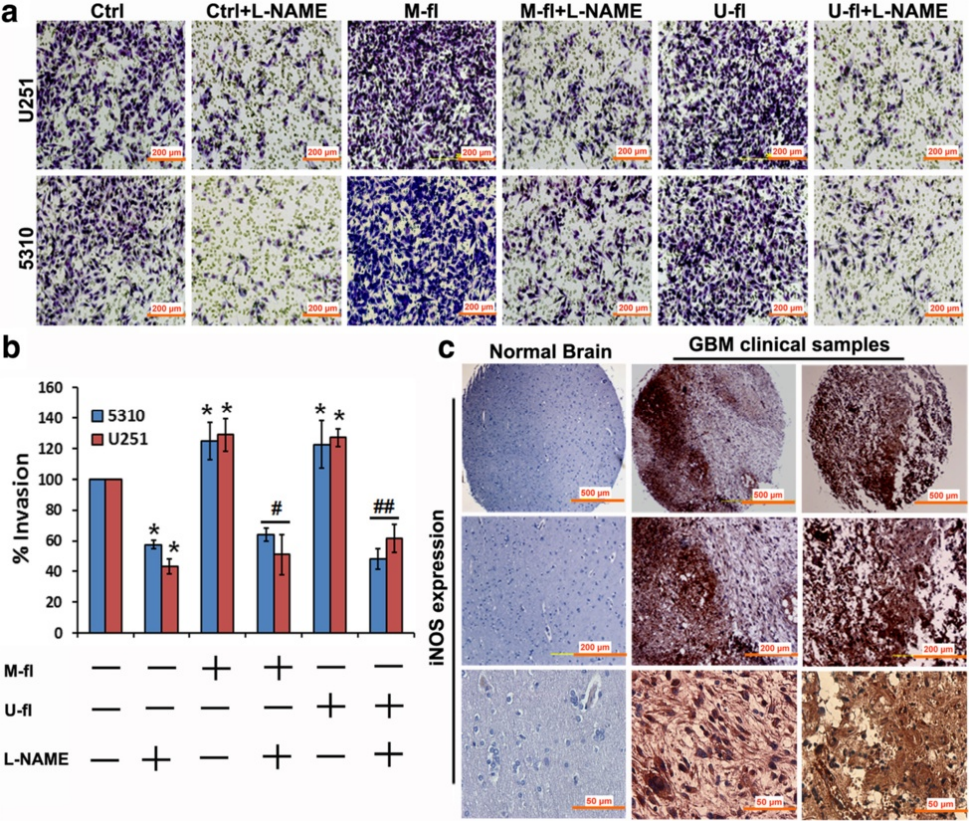
**Matrigel invasion assay of glioma cells and immunohistochemical analysis of glioblastoma clinical samples. (a)** Matrigel invasion assay of U251 and 5310 cells transfected with full-length MMP-9 (M-fl), and uPAR (U-fl) plasmids and treated with or without L-NAME. **(b)** Percent invasion was calculated from the mean of the average number of invaded cells obtained from three separate experiments. Columns represent mean (n = 3). Error bars represent ± SEM. *p < 0.05 vs. control. ^#^*p* < 0.05 vs. M-fl. ^##^*p* < 0.05 vs. U-fl. **(c)** GBM tissue microarrays were processed for immunohistochemical analysis followed by DAB staining to determine the presence of iNOS.

Recently, it was reported that treatment with NO donor, sodium nitroprusside significantly induced motility of glioma cell lines [17]. In addition application of the iNOS inhibitor, L-NAME, to these glioma cell lines impaired their movement. In the present study, prominent and significant reduction in wound healing (indicative of decreased migration potential) was noticed in L-NAME-treated control, M-fl-, and U-fl- transfected U251 glioma cells as compared to untreated cells from the respective groups (Figure 1a). In addition, our results have clearly demonstrated that the wound healing significantly increased (indicative of increased cell migration) in M-fl- and U-fl- transfected U251 glioma cells as compared to control U251 cells. This is in agreement with our earlier report wherein we showed an increased cell migration of 5310 human glioma xenograft cells after MMP-9 or uPAR overexpression [8]. Further, in the present study, we assessed the effect of iNOS inhibition on MMP-9- or uPAR-mediated glioma cell migration in U251 cells by spheroid migration assay. We noticed a significant reduction in the migration potential of M-fl- or U-fl- transfected U251 cells from their spheroids after treatment with L-NAME (Figure 1b). These results have clearly demonstrated the involvement of iNOS in the cell migration mediated by MMP-9 or uPAR in glioma cells. As expected, we noticed an increased invasion potential of both U251 glioma cells and 5310 glioma xenografts after transfection with M-fl and U-fl overexpression plasmids (Figure 2a). L-NAME treatment prominently and significantly reduced the invasion potential of untreated and M-fl- or U-fl- transfected U251 and 5310 cells (Figure 2b). In the present study, reduced invasion potential of untreated glioma cells after L-NAME treatment was also attributed to MMP-9 and uPAR involvement because simultaneous knockdown of MMP-9 and uPAR in glioma xenograft cells significantly reduced their invasion potential compared to untreated glioma cells [8].

### Inducible nitric oxide synthase expression in glioma

**Figure 3:**
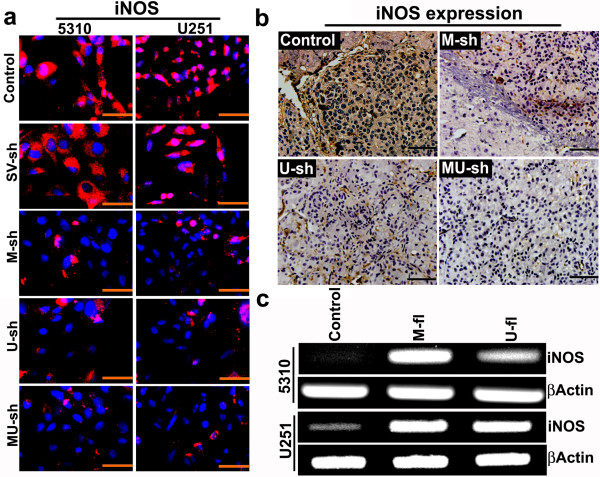
**Effect of various treatments on iNOS expression in glioma cells *in vitro *and *in vivo*. (a)** U251 and 5310 cells were transfected with scrambled vector (SV-sh), MMP-9 plasmid shRNA (M-sh), uPAR plasmid shRNA (U-sh), or MMP-9 + uPAR plasmid shRNA (MU-sh) and then subjected to immunocytochemical analysis for iNOS expression. **(b)** Immunohistochemical comparison of the iNOS expression in control, M-sh-, U-sh- and MU-sh-treated nude mice that were pre-injected (intracerebrally) with 5310 cells (0.2 × 10^6^ cells). **(c)** RT-PCR analysis of U251 and 5310 cells transfected with full-length MMP-9 (M-fl), and uPAR (U-fl) plasmids to evaluate the changes in iNOS mRNA expression.

Endogenous NO exhibits pleotropic roles within cancer cells and tumors, and studies employing inhibition or genetic deletion of endogenous NO synthases (NOSs) support a tumor-promoting role for NO [18,19]. We noticed prominent iNOS protein expression in clinical GBM samples (Figure 2c). We also noticed prominent iNOS expression in U251 and 5310 human glioma cells that were utilized in the present study (Figure 3a). High iNOS expression correlates with decreased survival in human glioma patients, and iNOS inhibition slows glioma growth in animal models [20]. MMP-9 or uPAR knockdown by shRNA-mediated gene silencing reduced iNOS protein expression in U251 and 5310 glioma cells. Reduction of iNOS expression was prominent when these cells were simultaneously downregulated with both MMP-9 and uPAR compared to their individual knockdowns (Figure 3a). Alternatively, it is also possible that the NO generated from iNOS activation can regulate both the expression of MMP-9 and its activation through cGMP dependent or independent mechanisms [11,12,21]. As expected, iNOS protein expression was noticed in gliomas obtained after intracranial implantation of 5310 cells in nude mice. However, these glioma cells-implanted nude mice showed reduced iNOS expression after treatments with M-sh, U-sh or MU-sh (Figure 3b). Recently, we have reported a significant reduction of intracranial tumor growth in these nude mice after M-sh, U-sh or MU-sh treatments [8,22]. Increased iNOS mRNA expression in MMP-9 or uPAR overexpressed glioma cells further demonstrated the interaction between MMP-9/uPAR and iNOS (Figure 3c).

### Interactions among MMP-9/uPAR, α9β1 integrin and iNOS in glioma cells

**Figure 4:**
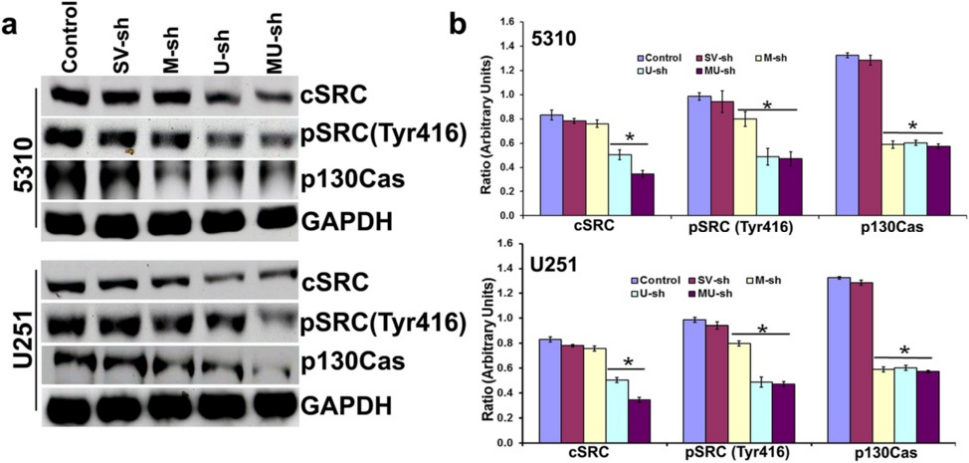
**Western blot analysis of U251 and 5310 glioma cells subjected to various treatments. (a)** Western blot analysis showing the effect of transfections with scrambled vector (SV-sh), MMP-9 plasmid shRNA (M-sh), uPAR plasmid shRNA (U-sh), or MMP-9 + uPAR plasmid shRNA (MU-sh) on the expression levels of several proteins associated with α9β1-mediated cell migration in U251 and 5310 glioma cells (n = 3). **(b)** Quantification of Western blot analysis results using Image J software. Columns represent mean (n = 3). Error bars represent ± SEM. *p < 0.05 vs. control.

**Figure 5:**
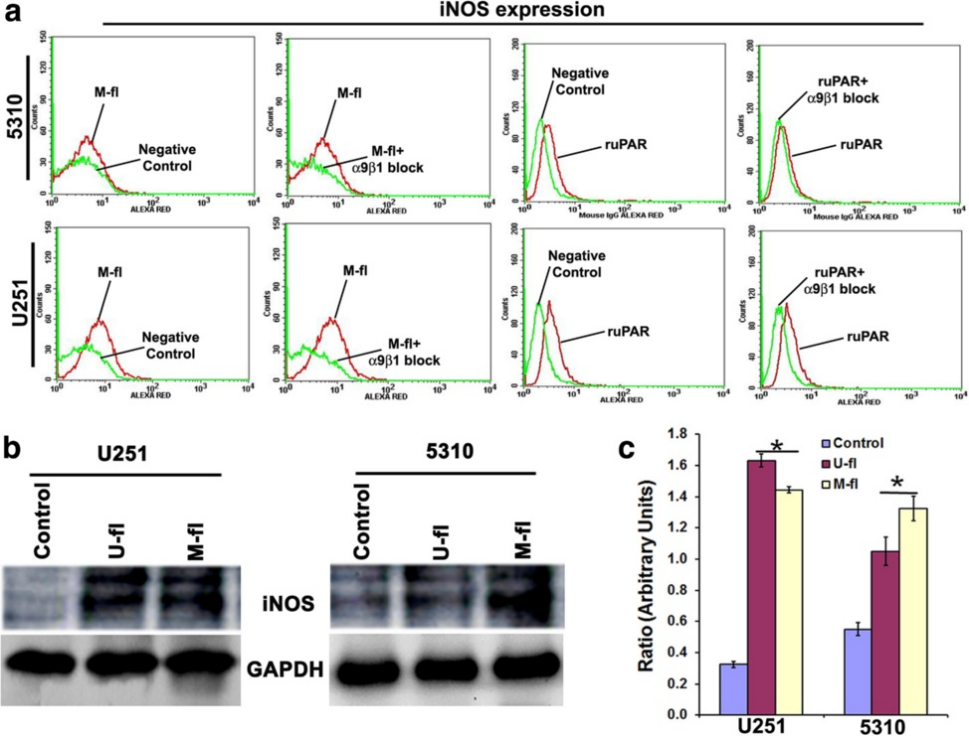
**FACS analysis and Western blot analysis. (a)** FACS analysis was performed to evaluate the effect of various treatments on iNOS expression in U251 glioma cells and 5310 glioma xenografts. **(b)** Western blot analysis showing the effect of various treatments on iNOS protein expression in U251 and 5310 cells. **(c)** Quantification of Western blot analysis results using Image J software. Columns represent mean (n = 3). Error bars represent ± SEM. *p < 0.05 vs. control.

**Figure 6:**
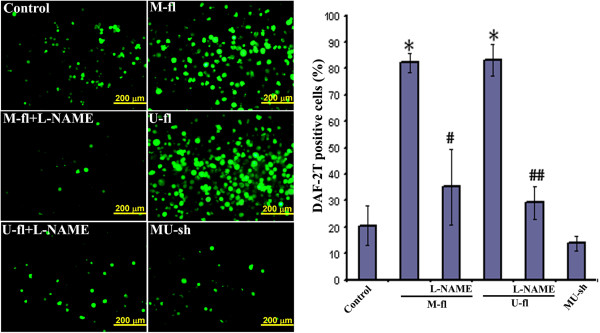
**Fluorescence microscopy of the DAF-2DA-loaded 5310 cells subjected to various treatments.** Representative images showing green fluorescence after transfection of 5310 glioma cells with full-length MMP-9 (M-fl) or uPAR (U-fl) plasmids, or MMP-9 + uPAR plasmid shRNA (MU-sh) followed by DAF-2DA treatment. Separate groups 5310 of cells transfected with M-fl or U-fl were treated for 4 hours with L-NAME, an inhibitor of iNOS at 1 mM concentration prior to DAF-2DA treatment. Bar graph represents the quantification of DAF-2 T positive 5310 glioma cells after various treatments (n = 3). Error bars represent ± SEM. *p < 0.05 vs. control. ^#^*p* < 0.05 vs. M-fl. ^##^*p* < 0.05 vs. U-fl.

Our recent studies clearly demonstrated the role played by α9β1 integrin in MMP-9-/uPAR-mediated glioma cell migration [8,23]. α9β1 integrin ligation can activate signaling through Src and FAK-mediated tyrosine phosphorylation of multiple proteins including p130Cas and paxillin [24,25]. In agreement with these reports, protein expression of several molecules [cSRC, pSRC (Tyr416), p130Cas] associated with α9β1-mediated cell migration were significantly affected after M-sh, U-sh, or MU-sh treatments in both U251 and 5310 cells (Figure 4a & 4b). Src activation was a proximal and dominant signaling regulating α9β1-mediated cell migration [25]. However, the molecular details of α9β1-induced Src activation remain to be elucidated. It could be possible that Src may directly interact with the cytoplasmic tail of α9, subsequently recruiting other signaling proteins to form an associated multimeric signaling complex which can activate iNOS. Recently it was shown that integrin α9β1 regulates iNOS activity via Src tyrosine kinase, resulting in increased NO production and NO-induced cell migration [25]. FACS analysis demonstrated that the overexpression of MMP-9 by transfection with MMP-9 overexpressing plasmid or treatment with recombinant uPAR in both U251 and 5310 glioma cells increased iNOS expression (Figure 5a). The increased iNOS expression in these cells has been reverted with α9β1 integrin blockade, indicating that MMP-9 or uPAR regulates iNOS via α9β1 integrin. Although the α9β1 integrin blockade in recombinant uPAR treated 5310 glioma cells did not prominently effect the iNOS expression, blockade of iNOS expression by L-NAME in uPAR overexpressed 5310 cells significantly reduced their invasion potential (Figure 5a & 2b). Further, α9β1 integrin blockade in uPAR overexpressed 5310 glioma cells significantly reduced their migration potential [8]. As expected, protein expression of iNOS was significantly increased upon MMP-9/uPAR overexpression in these glioma cells (Figure 5b & 5c). In addition to the reduced cell migration after L-NAME treatment in MMP-9 or uPAR overexpressed U251 glioma cells in the present study, increased NO production in MMP-9 or uPAR overexpressed glioma cells and the associated reduction in NO levels in those cells after L-NAME treatment clearly demonstrated the possible involvement of NO in MMP-9 or uPAR- regulated glioma cell migration (Figure 6). NO production was reduced in MMP-9 and uPAR knockdown 5310 glioma cells compared to controls (Figure 6). In the present study, although the reduced NO levels in MMP-9 and uPAR knockdown glioma cells are not significant compared to controls, the reduction in NO levels could be sufficient to significantly reduce glioma cell migration. These results allowed us to attribute the involvement of iNOS pathway in addition to other demonstrated pathways to the reduced glioma cell migration after MMP-9 and uPAR shRNA-mediated gene silencing that was demonstrated earlier [8].

**Figure 7:**
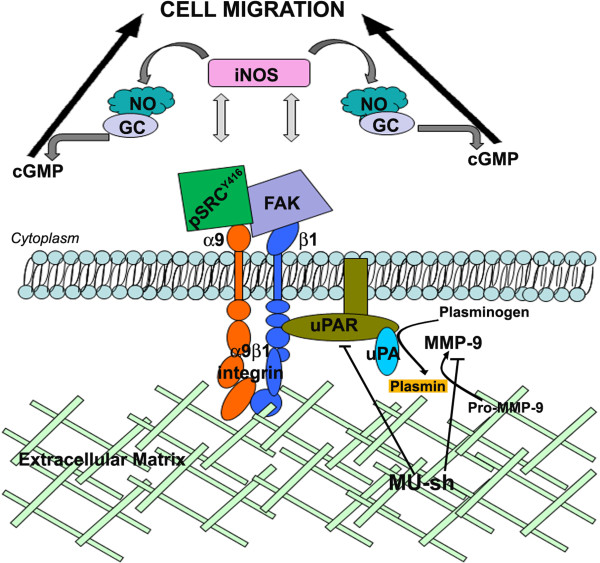
**Schematic representation of MMP-9- and/or uPAR-mediated glioma cell migration that utilizes the α9β1-iNOS pathway.** In glioma cells, uPAR and MMP-9 upregulate iNOS levels via their interactions with α9β1 integrin, which contributes to glioma cell migration. MU-sh treatment reduced α9β1 integrin levels and, in turn, reduced iNOS, an enzyme that produces NO.

Activation of iNOS can promote cancer cell migration via multiple mechanisms. NO generated from iNOS activation can act as a co-factor to GC to promote synthesis of the second messenger cGMP, which regulates cell migration in both a PKG dependent and independent fashion [11,12]. Relevant to integrin function, NO released into the cellular microenvironment can impact the assembly of focal adhesions. NO-induced delay of focal adhesion assembly or their premature de-stabilization has significant effects on cell migratory responses. Further, the reduced NO levels after inhibition of iNOS by genetic and pharmacological approaches impede glial cell proliferation, invasiveness, and tumor growth *in vivo *[26]. A previous study demonstrated that the natural products with anti-inflammatory effects such as wogonin and quercetin inhibited MMP-9 activity, iNOS expression and NO production in rat glioma C6 cells [27]. The reduced glioma cell migration in the present study after MMP-9 and/or uPAR knockdown is possibly attributed to the regulation of iNOS pathway via α9β1 integrin which are downstream to both MMP-9 and uPAR (Figure 7).

## Conclusions

MMP-9/uPAR overexpression enhanced the potential of glioma cell migration and invasion. L-NAME, an inhibitor of iNOS, inhibited MMP-9-/uPAR-induced glioma cell migration and invasion. iNOS expression was associated with GBM. MMP-9/uPAR overexpression increased iNOS expression and vice versa. MMP-9 and/or uPAR downregulation reduced the protein expression levels of several molecules associated with the α9β1-iNOS pathway mediated cell migration. In summary, glioma cells expressing MMP-9 and/or uPAR utilize α9β1-iNOS pathway to mediate cell migration.

## Abbreviations

MMP: Matrix metalloproteinase; UPAR: Urokinase plasminogen activator receptor; iNOS: Inducible nitric oxide synthase; NO: Nitric oxide; GBM: Glioblastoma multiforme; ECM: Extracellular matrix; PKG: Protein kinase G; L-NAME: L-N^G^-Nitroarginine methyl ester; GC: Guanylyl cyclase; cGMP: Cyclic guanosine monophosphate; RT-PCR: Reverse transcription polymerase chain reaction; PBS: Phosphate buffered saline; CMV: Cytomegalovirus; DAB: 3,3′-Diaminobenzidine; DAF-2DA: Diaminofluorescein-2 Diacetate.

## Competing interests

The authors declare that they have no competing interests.

## Authors’ contributions

JSR and KK Veeravalli were involved in the conception, hypotheses delineation, and design of the study. TZ conducted wound healing assay, spheroid migration assay, immunocytochemical, immunohistochemical and Western blot analysis. BC performed an assay that detects nitric oxide in cancer cells. SP performed Matrigel invasion assay, tissue array and RT-PCR analysis. CC involved in animal-related experiments. AAR and KK Velpula conducted FACS and Western blot analysis. The above-mentioned authors conducted the required experiments, performed the acquisition of the data or analyzed such information. BC and KK Veeravalli drafted the manuscript. EZ involved in the review of the manuscript prior to its submission. All authors read and approved the final manuscript.

## Pre-publication history

The pre-publication history for this paper can be accessed here:

http://www.biomedcentral.com/1471-2407/13/590/prepub
